# Electrochemical Analysis of Conducting Polymer Thin Films

**DOI:** 10.3390/ijms11041956

**Published:** 2010-04-26

**Authors:** Ritesh N. Vyas, Bin Wang

**Affiliations:** Department of Chemical Engineering, Lamar University, P.O. Box 10053, Beaumont, TX 77710, USA; E-Mail: riteshnvyas@gmail.com

**Keywords:** electrochemical impedance spectroscopy, cyclic voltammetry, layer-by-layer deposition, poly(*p*-phenylene vinylene), modified Randle’s circuit

## Abstract

Polyelectrolyte multilayers built via the layer-by-layer (LbL) method has been one of the most promising systems in the field of materials science. Layered structures can be constructed by the adsorption of various polyelectrolyte species onto the surface of a solid or liquid material by means of electrostatic interaction. The thickness of the adsorbed layers can be tuned precisely in the nanometer range. Stable, semiconducting thin films are interesting research subjects. We use a conducting polymer, poly(*p*-phenylene vinylene) (PPV), in the preparation of a stable thin film via the LbL method. Cyclic voltammetry and electrochemical impedance spectroscopy have been used to characterize the ionic conductivity of the PPV multilayer films. The ionic conductivity of the films has been found to be dependent on the polymerization temperature. The film conductivity can be fitted to a modified Randle’s circuit. The circuit equivalent calculations are performed to provide the diffusion coefficient values.

## 1. Introduction

An in-depth analysis of mass and/or charge transfer mechanism is highly desired for successful development in electrochemical devices. For example, in fuel cell catalyst applications, the catalyst layer deposited on a polyelectrolyte membrane would demand higher ionic (protonic) conductivity. In the electrochemical sensors, the overall performance depends largely upon sensitivity of the thin films to recognize the analyte and the speed to communicate the resultant signals with the underlying electrodes. These phenomena are closely related to the mass and/or charge transfer within such films. A clear understanding of mass transfer in such an electrochemical system is the prerequisite for major progress in devices.

**Figure 1.**
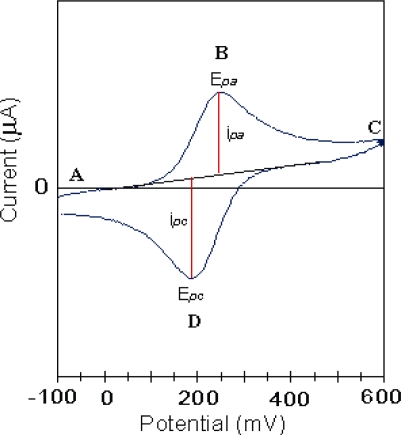
Schematic cyclic voltammogram for redox couple undergoing single electron oxidation-reduction process.

In an electrochemical cell with a conventional three-electrode set-up, suppose there is a redox couple undergoing a one electron oxidation-reduction process: **Ox** + e^−^ ↔ **R**. The cyclic voltammogram for such a process is shown in Figure 1. Points **B** and **D** describe the simultaneous oxidation and reduction processes respectively. As a result of these reactions a concentration gradient is developed at the surface of the electrode for both species. Thus, we obtain a ‘diffusion controlled mass transfer process’ of species **Ox** from the electrolyte to the surface of the electrode. In electrochemical studies, such a transport is termed as ‘ionic charge transfer’ or ‘mass transfer’ in the literature.

Fundamentally, three different types of mass-transfer phenomena exist for ionic species in electrolytes near the electrodes: (1) diffusional transport under concentration gradients, (2) migration transport of oppositely charged ions under electric field of the electrode, and (3) convection transport due to physical stirring of the electrolyte [1]. Here, the ‘electron transfer’ occurs between the redox couple (**O_x_** or **R**) and the electrode. However, for electrodes modified with electroactive or redox films (e.g., conducting polymer films), the redox behavior is more complicated. When the applied potential reaches the oxidation potential of the redox-active species in the film, the electron transfer from the electrode surface to the film is coupled with the simultaneous ionic transfer from electrolyte to the film for maintaining the electro-neutrality [2]. Thus, we observe two simultaneous mass-transfer processes at the same time and each one needs to be characterized individually. A detailed study of mass-transfer into such films would first require conceptual understanding of the main characterization techniques used. We will first discuss some basics of electrochemical impedance spectroscopy (EIS) application for the characterization of mass transfer in thin films, followed by the modeling for analyzing ionic mass-transfer in poly(*p*-phenylene vinylene) (PPV) films.

In the early 90s Decher and Hong [3] established the layer-by-layer (LbL) assembly method as a versatile technique to prepare thin polymer films. This self-assembly method initially involved sequential adsorption of alternating polyelectrolytes from dilute solution onto an oppositely charged substrate [4]. Many films constructed through the LbL method rely on the electrostatic interaction between the polyelectrolytes to maintain the structural integrity. Variable solution conditions, especially high ionic strength, can greatly alter or even destroy film structures [5–11]. Therefore, preservation of the film structures can be challenging for electrochemical applications of LbL films because electrochemical reactions often encounter high ionic strength solution conditions. Intuitively, a protective thin film deposited on top of the functional LbL structures seems a desirable solution to the film stability dilemma. Cross-linking is a common method to form stable polymeric structures. Photo-initiated [12–17], thermal-induced [18,19], and conventional chemical [20–22] reactions have been employed to produce cross-linked LbL films. During such reactions, a new covalent bond is formed between the constituent polyelectrolyte chains to produce an insoluble structure. The resultant, cross-linked films often become less permeable to ions in solution due to reduced ionic sites.

Stable conductive polymeric films are interesting research subjects. Oxidative cross-linking of thiophene monomers has produced conducting polymer coatings [23,24]. Fixation of carbon black onto particles is employed to prepare conductive composites [25] that may find use as thermal resistors, chemical sensors, and electrostatic dissipation layers. We choose poly (*p*-phenylene vinylene) to prepare LbL assembled conducting polymer films. PPV and its derivatives have been successfully used for light-emitting diodes [26], photovoltaics [27], and solid-state laser devices [28]. A water soluble, commercially available PPV precursor (*pre*-PPV) has been employed to prepare PPV thin films via the LbL method [29,30]. We have prepared PPV-containing thin films via the LbL method recently [31]. Thermal polymerization converts *pre*-PPV chains to highly intractable PPV, thus making the resulting films insoluble.

## 2. Experimental Section

*Chemicals*. Cysteamine hydrochloride (cyst), 3-aminopropyltriethoxysilane (APTES), and 12-phosphomolybdic acid hydrate (H_3_PMo_12_O_40_·nH_2_O) (commercially obtained from Sigma-Aldrich) were of reagent grade. Potassium ferricyanide (K_3_[Fe(CN)_6_]), potassium ferrocyanide (K_4_[Fe(CN)_6_]) and sodium phosphate were purchased from Malinckrodt as reagent grade and used without further purification. Polyethylenimine (PEI; MW 750,000; 50 wt % in water), poly(acrylic acid) (PAA; MW 100,000; 30 wt % in water), poly(sodium 4-styrenesulfonate) (PSS; MW 70,000; 50 wt % in water.), poly(diallyl dimethyl ammonium chloride) (PDDA; MW 250,000; 50 wt % in water) and poly(*p*-xylene tetrahydrothiophenium chloride) (*pre*-PPV; MW (repeat unit) 226.5; 0.25 vol % in water) were also purchased from Aldrich and prepared with nanopure water to desired solutions (PEI, 0.01 M, pH 6.5; PAA, 0.001 M, pH 3.5, 0.1 M NaCl; PSS, 0.001 M, pH 3.5, 0.1 M NaCl; *pre*-PPV, 1 × 10^−5^ M). Other solution parameters are explained in Table 1. The aqueous solutions of PDDA were centrifuged for 20 minutes before use while the *pre*-PPV solution was filtered through a 0.45 μm filter. All water used was purified by Barnsted Nanopure II purification system. The resistivity was about 18 MΩ/cm.

*Substrates*. Indium-tin-oxide (ITO) coated glass slides (Sigma-Aldrich, 80–100 Ω) and gold coated glass slides (Bioanalytical Systems) were used for cyclic voltammetry and electrochemical impedance spectroscopy. ITO slides were cleaned as follows: the slides were first sonicated for 20 min each in acetone, methanol and water. After cleaning, they were subjected to potential cycling (−0.2 V to 0.7 V, 50 mV/s) in 0.1 M HCl solution for 50 cycles (or until the characteristic ITO curve is obtained) to make the surface electrochemically active. Thereafter, the slides were cleaned with water, dried under a stream of air and dipped in APTES (2 v % methanol) solution for 24 h. Subsequently, the slides were washed by consecutive dipping in three water baths for 3 min each and dried with a gentle stream of air immediately before the multilayer deposition.

Gold coated glass slides were cleaned by dipping them in a solution containing three parts H_2_SO_4_ (96 wt %) and one part H_2_O_2_ (30 wt %) at room temperature for 20 min followed by three consecutive sonications in water for 10 min and potential cycling (0 V–1.7 V, 50 mV/s) in 2 M H_2_SO_4_ solution. The slides were then rinsed with water and dipped in aqueous solution (0.02 M) of cyst for 24 h. After this treatment, the slides were washed by consecutive dipping in three water baths for 3 min each and dried with a gentle stream of air immediately before the multilayer deposition.

*Film Preparation*. Table 1 summarizes the preparation conditions for fabricating [cyst-(PPV│PSS)_3_] (samples **I**–**III**), [cyst-(*pre*-PPV│PSS)_3_] (sample **IV**), cyst-(PPV│PAA)_3_ (sample **V**), [APTES-{(PMo_12_│PDDA)_10_-(PSS│PPV)_3_}] (sample **VI**) and [APTES-(PMo_12_│PDDA)_10_] (sample **VII**) films. For preparing samples **I**–**IV**, cyst-coated gold slides were dipped in negatively charged PSS solution for 10 min followed by three consecutive wash steps as follows: (i) 20 dips each of 5 s in water, (ii) five dips each of 20 s in water, and (iii) a 3 min dip in continuously agitated water. The substrates were then dipped in positively charged *pre*-PPV solution for 10 min followed by the same wash sequence. The entire cycle was repeated for three consecutive times to yield cyst-coated gold substrate with three bilayers of (PSS) and (*pre*-PPV) that can be written as (*pre*-PPV│PSS)_3_. After deposition, the prepared films were heated under vacuum at three different conditions: (i) 110 °C for 2 h [32], (ii) 180 °C for 4 h [33], and (iii) 210 °C for 12 h [29–31], where the heating conditions were chosen following the literature methods. The (PPV│PSS) 3 samples prepared according to conditions (i), (ii) and (iii) are termed **I**, **II** and **III** respectively while sample **IV** is a non-heated (*pre*-PPV│PSS)_3_ film used as a control. Sample **V** is similarly prepared (PPV│PAA) 3 film using the preparation conditions identical to sample **III** and replacing PSS during the LbL deposition with PAA.

For preparing samples **VI** and **VII**, first an APTES-coated ITO slide was coated with PMo_12_ and PDDA following the procedure described in literature [34]. After that, on top of this (PMo_12_│PDDA)_10_ film, a (PSS│ *pre*-PPV)_3_ film is deposited by repeating the procedure for preparing sample **III** as above, including thermal polymerization at 210 °C. This [(PMo_12_│PDDA)_10_-(PSS│PPV)_3_] film is termed as sample **VI**. The sample **VII** is a similar APTES-coated ITO slide having (PMo_12_│PDDA)_10_ bilayers without any (PSS│PPV)_3_ bilayers on the top.

*Apparatus.* The multilayer assembly was fabricated manually as well as with an R & K dipping robot (Ultrathin Organic Film Technology, Germany). Cyclic voltammetry measurements were conducted on a Voltalab 10 PGZ 100 Potentiostat equipped with VoltaMaster 4 Electrochemical Software version 2.10. A conventional three-electrode setup was used. The working electrode was the ITO or gold-coated glass slide modified with self-assembled films, while a platinum wire was used as the counter electrode. An Ag/AgCl (3 M KCl) reference electrode was used for all measurements and 0.1 M HCl was used as an electrolyte. Electrochemical impedance spectroscopy was performed within the frequency range of 1–10^5^ Hz with amplitude of the applied sine wave potential as 10 mV and applied dc potential as 220 mV. A mixture of 5 mM concentration of K_3_[Fe(CN)_6_] and K_4_[Fe(CN)_6_] (1:1) in 0.025 M sodium phosphate was used as the redox probes. The data of EIS experimental spectrum were fitted with Zview software. The atomic force microscopy (AFM) image was recorded on gold using a Quesant Q-Scope TM-250 instrument operating in the tapping mode, using a silicon cantilever unit (nanoprobe). The scanning frequency was set at 2 Hz. A drive amplitude of 10–15 mV was used.

## 3. Results and Discussion

### 3.1. Theoretical Background

A detailed study of mass-transfer phenomena in thin films requires conceptual understanding of the main characterization techniques used. In case of conducting polymer films, because of the electrochemically active nature of the polymer itself, highly versatile techniques are preferred to accommodate the complex processes involved. One such technique well suited for such characterization is the electrochemical impedance spectroscopy. Traditionally, EIS was applied to study AC polarography and in determining double layer capacitance at the electrodes [35]. Currently, EIS has been widely used as a versatile technique to characterize interfacial and transport properties of polymer films, organic-inorganic coatings, and self-assembled monolayers adsorbed on the surface of an electrode [36]. The estimation of parameters such as diffusion coefficient and charge transfer resistance that can explain the kinetics at interfaces becomes relatively easy and accurate as compared to other techniques [36]. Basically, a small amplitude AC signal is imposed on the system under study and the impedance measurements are taken at various frequencies of the applied AC signal [37].

Electro-active polymers have been extensively studied in last two decades because of their potential applications such as in electrocatalysis, energy storage, and sensors. The study for electro-active polymers fall under two major categories in the literature: redox and conducting polymers. Redox polymers are considered as an electrochemically inert polymer network, where the redox centers are covalently bound and each center is assumed immobile such that there is no direct chemical bond with other redox centers [38,39]. For electrochemically active conducting polymers, the overall process of oxidation-reductions with doping/undoping of polymer is considered as delocalization of charges and unpaired electrons over a large number of monomer units [38]. In the current paper, we will discuss the electrochemical aspects of conducting polymer films constructed via the LbL deposition method.

**Figure 2.**
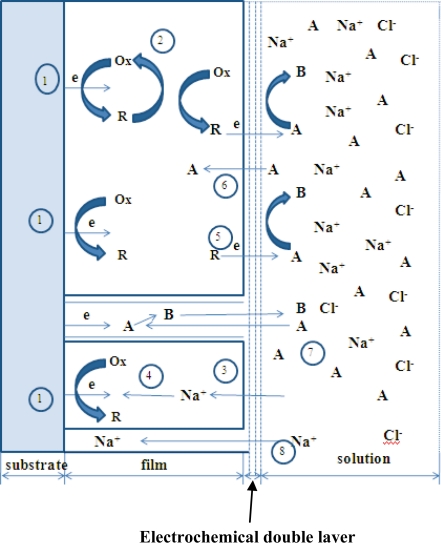
Summary of processes that may occur at electrodes modified with conducting polymers in presence of redox-couple in electrolyte.

EIS studies have been carried out on polypyrrole [40], polyaniline [41] and poly(3,4 ethylenedioxythiophene) [42] films in presence of a redox-active electrolyte. Electro-neutrality within the polymer film is essential during the reversible redox reaction of conducting polymers and is facilitated by the movement of ions between the polymer and the electrolyte solution. Overall, this process includes electron transfer at electrode-film interface, electron and counter ion-transport (e.g., Na^+^ and Cl^−^ ions in case electrolyte used is dilute NaCl solution) in the conducting polymer film and diffusion redox-ion transfer (generally linear diffusion) at film-electrolyte interface (Figure 2).

However, these interfaces are different from the ones that occur in redox polymer films since electron transfer can also occur at the film-electrolyte interface due to the redox couple present in the solution (Process 5) [41,42]. Thus, we can observe two parallel processes if the oxidation-reduction potential of the redox-couple falls within the potential range where the conducting polymer is in its oxidized-reduced or conducting state. Let us assume that species **Ox** is an electroactive species in the film getting reduced to **R** at the film-electrolyte interface: **Ox** + e^−^ ↔ **R.** For the sake of simplicity, let us consider NaCl as electrolyte and species **A/B** represent a redox couple (e.g., Fe(CN)_6_^3−/4−^) in the solution. Figure 2 summarizes the processes that may occur for an electroactive film in the presence of a redox couple:

1. Heterogeneous electron transfer to **Ox** to produce reduced form **R.**
2. Electron transfer from **R** to another **Ox** in the film (electron diffusion or electron hopping in the film).
3. Ionic diffusion of Na^+^/Cl^−^ from solution into the film to maintain electro-neutrality.
4. Ionic transfer (conduction) of Na^+^/Cl^−^ within the film.
5. Electron transfer from **R** to **A** at film/solution interface to form **B**.
6. Mass transfer (linear diffusion) of **A** into the film under concentration gradient.
7. Movement (migration) of **A** through a pinhole or channel in the film to substrate where it can be reduced.

**Figure 3.**
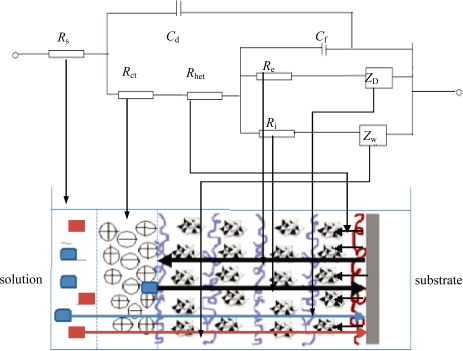
Equivalent circuit and corresponding model described in Figure 2.

Equivalent circuit as shown in Figure 3 is carefully constructed to understand each process individually. The charge transfer resistance *R*_het_ is used to explain resistance to overall heterogeneous transfers of electrons at metal-film and film-electrolyte interfaces (process 1). *R*_e_ and *R*_i_ explain the real resistances to electronic and ionic diffusion within the film as described by process 2 & 4 respectively. The diffusion of counter-ions from electrolyte towards the electrode surface (process 7) is described by Warburg element *Z*_D_. The diffusion of redox couple under concentration gradient is considered semi-infinite planar (process 6) and the resistance to this diffusion is given by Warburg element *Z*_w_.

**Figure 4.**
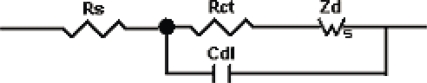
A conventional Randle’s circuit. *R*_s_ is solution resistance, *R*_ct_ is charge transfer resistance, *Z*_d_ is the Warburg impedance, and *C*_dl_ is the double layer capacitance.

The circuit seems analogous to the equivalent circuit used to explain the electrochemical behavior of conducting polymer films of poly(3,4 ethylenedioxythiophene) (PEDOT) as explained by others [42]. For these films, two simultaneous processes (*i.e.*, diffusion of electrolyte ions and electrons inside the films as well as the charge transfer at film-electrolyte interface due to redox active species) are expected because redox potential used in the experiments fall in similar range (PEDOT = 0.1–0.2 V [35] and Fe(CN)_6_)^3−4−^ = 0.22 V). However for thin films of conducting polymers like PPV in the presence of a redox couple like (Fe(CN)_6_)^3−4−^, we cannot have two simultaneous processes because their redox potentials do not fall in the same range (PPV = 0.6 V [43]). Thus, for applied potential of 0.22 V, we can only expect the diffusion of ferrocyanide ions into the PPV films to be a primary diffusion mechanism. At these experimental conditions, the equivalent circuit in above figure could be modified into a very simple form as shown in Figure 4.

**Figure 5.**
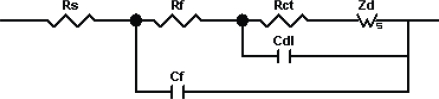
A modified Randle’s equivalent circuit. *R*_s_ is solution resistance, *R*_ct_ is charge transfer resistance, *R*_f_ is the film resistance, *Z*_d_ is the Warburg impedance, *C*_f_ is the film capacitance, and *C*_dl_ is the double layer capacitance.

*R*_f_ (*R*_i_ in previous case) represents the resistance to the ionic diffusion of ferrocyanide ions into the film and *R*_ct_ is the charge transfer at the film-electrolyte interface. *Z*_w_ represents the Warburg diffusion parameter for the ionic diffusion. Note that the circuit is very similar to an ideal Randle’s circuit commonly used to characterize thin films as shown in Figure 5.

The real and imaginary values for impedance for an ideal Randle’s circuit (Figure 4) can be given as [36]:

(1) $${\left(Z_{re}\right)}_{1}=R_{s}+\frac{R_{ct}+{\sigma \omega }^{1/2}}{{\left(C_{dl}{\sigma \omega }^{1/2}+1\right)}^{2}+\omega^{2}C_{dl}^{2}{\left(R_{ct}+{\sigma \omega }^{-1/2}\right)}^{2}}$$

(2) $${\left(Z_{im}\right)}_{1}=\frac{{\omega C}_{dl}{\left(R_{ct}+{\sigma \omega }^{-1/2}\right)}^{2}+{\sigma \omega }^{-1/2}\left(C_{dl}{\sigma \omega }^{1/2}+1\right)}{{\left(C_{dl}{\sigma \omega }^{1/2}+1\right)}^{2}+\omega^{2}C_{dl}^{2}{\left(R_{ct}+{\sigma \omega }^{-1/2}\right)}^{2}}$$

where *ω* is the radial frequency and *σ* is the Warburg parameter. If the deposited film behaves as an ideal capacitor on the electrode, then we always observe a vertical line with a unity slope in low-frequency region [35]. For the high frequency region, the intercept with real impedance axis would also give the accurate values for solution resistance *R*_s_. However, the charge transfer resistance *R*_ct_ and double layer capacitance *C*_f_ only describe the resistance and capacitance provided by the electrochemical double layer at the interface.

In our study of functional LbL films [44], the experimental data show depressed semi-circles in high frequency region with a slight deviation from unity slope in lower frequency region. Thus, a resistance to the movements of redox ions inside and out of the deposited film needs to be addressed to the circuit along with the charge transfer resistance. Adding a film resistance (*R*_f_) in parallel to double layer capacitance in the circuit has been employed for self-assembled monolayer. Silva and coworkers dealt with this problem differently and employed two models in the case of polyelectrolyte films with increasing thickness [45,46]. Here, the Randle’s circuit was modified by addition of two more resistances, namely film resistance *R*_f_ offered by multilayers and *R*_m_ due to Ohmic conduction within the film. The films in their studies exhibited non-linear diffusion; in contrast, our films demonstrate semi-infinite diffusion patterns. Here, we adapt a similar Randle’s circuit as shown in Figure 5, but add two more elements to the circuit to define the properties of film: film resistance (*R*_f_) and film capacitance (*C*_f_). The Equations (1) and (2) are modified as

(3) $${\left(Z_{\text{re}}\right)}_{2}\;=\;R_{\text{s}}\;+\;\frac{\frac{R_{\text{f}}+{\left(Z_{\text{re}}\right)}_{1}}{{\left({\omega C}_{f}\right)}^{2}}}{{\left[R_{\text{f}}+{\left(Z_{\text{re}}\right)}_{1}\right]}^{2}+{\left[{\left(Z_{\text{img}}\right)}_{1}-\frac{1}{{\omega C}_{f}}\right]}^{2}}$$

(4) $${\left(Z_{\text{img}}\right)}_{2}\;=\;R_{\text{s}}+\frac{\frac{{\left(Z_{\text{img}}\right)}_{1}}{{\left({\omega C}_{f}\right)}^{2}}\;+\;\frac{{\left({\left(Z_{\text{img}}\right)}_{1}\right)}^{2}-{\left({\left(Z_{\text{re}}\right)}_{1}\right)}^{2}-R_{\text{f}}{\left(Z_{\text{re}}\right)}_{1}}{{\omega C}_{f}}}{{\left[R_{\text{f}}+{\left(Z_{re}\right)}_{1}\right]}^{2}+{\left[{\left(Z_{\text{img}}\right)}_{1}-\frac{1}{{\omega C}_{f}}\right]}^{2}}$$

where (*Z*_re_)_2_ = *Z*’ is the real component of the modified Randle’s circuit, and (*Z*_re_)_2_ = *Z*” is the imaginary component of the circuit. After modifying the circuit, we can develop equations to calculate the diffusion coefficients for thin film samples. At low frequencies (*ω* → 0), Equations (3) and (4) become:

(5) $$Z^{\prime }=R_{\text{s}}+R_{\text{f}}+R_{\text{ct}}+{\sigma \omega }^{-1/2}$$

(6) $$Z^{\prime \prime }={\sigma \omega }^{-1/2}+{2\sigma }^{2}C_{\text{dl}}+4\sigma^{4}C_{\text{dl}}^{2}C_{\text{f}}-R_{\text{f}}^{2}C_{\text{f}}-R_{\text{ct}}^{2}C_{\text{f}}$$

From Equation (5), the plot of *Z’ vs*. *ω*^−½^ gives slope = *σ* and intercept = (*R*_s_ + *R*_f_ + *R*_ct_). The intersection of a Nyquist plot with x-axis gives the values for solution resistance *R*_s_ that can be used to calculate *R*_ct_ from the intercept values. The Warburg parameter *σ* and diffusion coefficient *D* can be obtained according to [35]:

(7) $$\sigma =\frac{\text{RT}}{\sqrt{2}n^{2}F^{2}A}\left(\frac{1}{D_{\text{Ox}}^{1/2}c_{\text{Ox}}}+\frac{1}{D_{\text{red}}^{1/2}c_{\text{red}}}\right)$$

where *n* is the number of electrons transferred (in this case 1), *F* is Faraday’s constant (96485 C·mol^−1^), *A* is the electrode area (1 cm^2^), *R* is gas constant (8.314 J·mol^−1^·K^−1^) and *T* is room temperature (298 K). Assuming diffusion coefficients *D*_ox_ = *D*_red_ = *D* and concentrations *c*_ox_ = *c*_Red_ = *c*_bulk_ we get [42]:

(8) $$D={\left(\frac{\sqrt{2}\text{RT}}{n^{2}F^{2}{A\sigma c}_{\text{bulk}}}\right)}^{2}$$

From Equation (8), the calculated values for diffusion coefficients become approximation values and are called apparent diffusion coefficients.

### 3.2. Electrochemical Analysis of Poly(*p*-phenylene vinylene) Films

**Figure 6.**
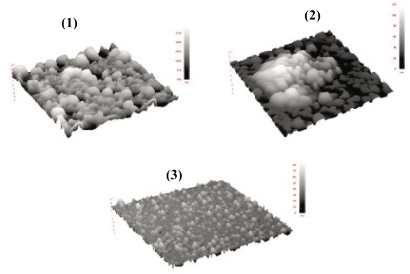
(5 μm × 5 μm) Tapping mode AFM images of **(1)** (PPV│PSS)_1_, **(2)** (PPV│PSS)_2_ and **(3)** (PPV│PSS)_3_ films.

We recently prepared thin films of poly(*p*-phenylene vinylene) via the LbL method [31]. Thermal polymerization converts *pre*-PPV chains to highly intractable PPV, thus making the resulting films insoluble. These electronically conducting films can also be used as a conductive stable layer to cover an electroactive film such that the overall conductivity of the film is not compromised. We choose two anionic polyelectrolytes, poly(styrene sulfonate) (PSS) and poly(acrylic acid) (PAA), as the counter polyelectrolyte to complement *pre*-PPV in the LbL preparation. A (PPV│PSS) or (PPV│PAA) film with higher number of bilayers is speculated to offer higher stability to the inner film. But thicker films possess increased resistance so the conductivity of the films will be compromised. On the other hand, using a lesser number of bilayers would help in providing electron transfer but stability of the film will be compromised. Thus, we first determine the number of (PPV│PSS) or (PPV│PAA) bilayers required to provide full coverage on an electrode surface by using atomic force microscopy (AFM). Figure 6 shows the three-dimensional AFM images (5 μm × 5 μm) for one, two and three bilayers of (PPV│PSS) on gold slides prepared by our previously published procedures [31]. We clearly observe incomplete, partial and complete coverage of the film for one, two and three bilayers, respectively. Similar results were obtained for one, two and three bilayers of (PPV│PAA) film (figures not shown here).

Thereafter, our experiments were focused on films with three bilayers. The selection of (PPV│PSS)_3_ over (PPV│PAA) 3 was done with EIS measurements as explained later in this section. Thus, four different (PPV│PSS) 3 films were prepared initially for EIS characterizations. Table 1 summarizes preparation conditions for fabricating [cyst-(PPV│PSS)_3_] (samples **I**–**III**), [cyst-(*pre-*PPV│PSS)_3_] (sample **IV**), [cyst-(PPV│PAA)_3_] (sample **V**), [APTES-[(PMo_12_│PDDA)_10-_(PSS│PPV)_3_] (sample **VI**) and [APTES-(PMo_12_│PDDA)_10_] (sample **VII**) films.

**Figure 7.**
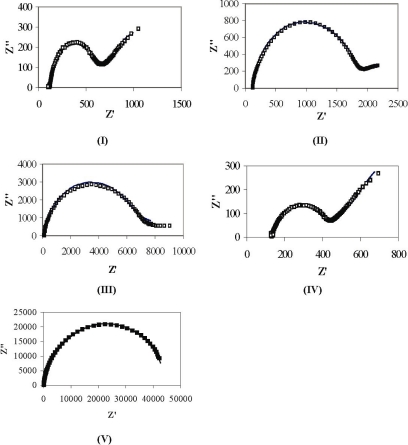
Impedance spectra (0.005 M Fe(CN)_6_^3−/4−^ in 0.025 M Na_2_HPO_4_, pH 6.3) for gold electrodes with (PPV│PSS) 3 (Samples **I-III**), (*pre*-PPV│PSS)_3_ (Sample **IV**) and (PPV│PAA)_3_ (Sample **V**) multilayer assembly. Note: (□), experimental; (−), theoretical fittings using the parameter values in Table 2. Frequency range, 1- 10^5^ Hz; Sinusoidal voltage, 10 mV; dc potential, 220 mV.

After polymerization, the samples **I**–**V** were subjected to EIS analysis. The Nyquist plots of (PPV│PSS)_3_ films deposited on gold are shown in Figure 7. The *Z*’ and *Z*” axes are not drawn to scale in order to artificially emphasize the so-called semi-circle characteristic of the curves. The semi-circle at higher frequencies and a straight line with unity slope (Warburg line) at lower frequencies can be considered as a characteristic of linear diffusion [1,46]. With an increase in conversion temperature (sample **I**–**III**) the diameter of the semi-circle is observed to increase, indicating higher resistance to mass transfer. Moreover, Warburg slope diminishes partially (sample **II**) and then completely (sample **III**) indicating the insulating properties (partial and complete drop in diffusion coefficients) of the film for sample **II** and **III** respectively.

The quantitative analysis of film resistance for sample **I**–**V** shows a much clearer picture. An equivalent circuit, as shown in Figure 5, has been used to collect quantitative information about different electrochemical parameters defining the process [18]. The elements *R*_f_ and *C*_f_ represent the multilayer resistance and capacitance respectively and generally depend on film thickness, ion content and mobility of ions [47]. *C*_dl_ accounts for the double layer capacitance at the surface of the film while *R*_ct_ is the charge transfer resistance for the redox phenomenon occurring at the film-electrolyte interface. *Z*_d_ accounts for the Warburg impedance due to the mass transfer of the redox species to the electrode as represented by Warburg slope, and *R*_s_ is the solution resistance. The equivalent circuit provides an excellent fit (<5% error) to the experimental data as summarized in Table 2 [48]. Compared to sample **IV**, increases in values of *R*_f_ by a factor of 1.6, 6.5 and 20, and *R*_ct_ by a factor of 1.5, 4.8, and 17 were observed for samples **I**, **II**, and **III**, respectively. The EIS analysis result of a sample **V** demonstrated high film resistance (20 KΩ·cm^2^) and charge transfer resistance (24 KΩ·cm^2^).

The increases in *R*_f_ and *R*_ct_ in samples **I-III** as compared to sample **IV** can be explained by an increase in glass transition temperature *T*_g_ with increasing polymerization temperatures [49]. The highest polymerization temperature condition employed for the preparation of sample **III** does produce the film with the highest resistance. However, when compared to the *R*_f_ and *R*_ct_ values of the non-conducting polymer films reported [18], sample **III** was about 35 times more conductive. One explanation to this is that PSS can induce *p*-type doping of PPV chains inducing higher hole transfer rate and eventually more conductivity than PAA [31]. For a similar film with PAA (sample **V**), these values are about 8 times less conductive than sample **III**. Thus, LbL films of PPV and electronically conducting nanoparticles can be imagined to produce stable layers with even higher conductivity.

**Figure 8.**
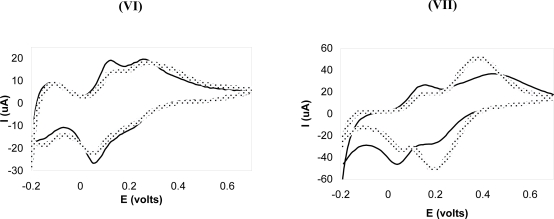
Cyclic voltammograms before (–––) and after (----) 500 Cycles for Samples **VI** and **VII**. Note: scan rate, 0.2 V·s^−1^; electrolyte, 0.1 M HCl; reference electrode, Ag/AgCl (3 M KCl); counter electrode, Pt wire; working electrode, LbL-coated ITO.

**Figure 9.**
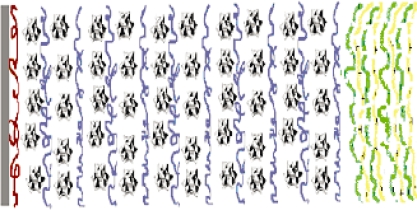
Schematic diagram for [(PMo_12_│PDDA)_10_-(PPV│PSS)_3_] films. Legends: gray slab, substrate; brown chains, PEI; polyhedrons, PMo_12_; blue chains, PDDA; yellow chains, PPV; green chains, PAA/PSS.

We use PMo_12_-containing LbL structure to test the stability of the (PPV│PSS) films. PMo_12_ clusters are well soluble in water [50], so stabilization of their LbL films can be a challenge. Figure 8 demonstrates the results for samples **VI** and **VII** in Table 1. Without protection, dissolution of POM containing LbL films in high ionic strength aqueous solutions has been observed in sample **VII**. When the (PMo_12_│PDDA)_10_ film was protected by a (PPV│PSS) 3 layer, the [(PMo_12_│PDDA)_10-_ (PSS│PPV)_3_] (sample **VI**) film nicely resembled the original curve. Figure 9 shows a schematic of the (PSS│PPV)_3_ film used as a protection layer for (PMo_12_│PDDA)_10_ assembly.

## 4. Conclusions

In summary, we have built conducting polymer layer-by-layer films that show electrochemical activities. To analyze the properties of such films, a modified Randle’s circuit equivalent was developed to calculate a redox species diffusion through the conducting polymer thin films. By comparing the electrochemical analysis results, namely EIS and CV results, it was shown that polymerization temperature to be the determining factor of the film properties. After heating at 210 °C overnight, the produced films become highly stable and less conductive. The layered structure can be used to preserve nanoparticle films against potential cycling in a high ionic strength solution.

## Figures and Tables

**Table 1.** Preparation parameters used in layer-by-layer construction: only pss used with salt.

**Sample**	**Polymerization condition**	***p-*PPV (mM)**	**PAA (pH)**	**PSS (pH)**	**NaCl (M)**	**PMo_12_ (mM)**	**PDDA (mM)**
**I**	110 °C @ 2 h	0.1	-	4.0	0.1	-	-
**II**	180 °C @ 4 h	0.1	-	4.0	0.1	-	-
**III**	210 °C @ 12 h	0.1	-	4.0	0.1	-	-
**IV**	No heating	0.1	-	4.0	0.1	-	-
**V**	210 °C @ 12 h	0.1	4.0	-	0.1	-	-
**VI**	210 °C @ 12 h	0.1	-	4.0	0.1	5	10
**VII**	No heating	-	-	-	-	5	10

**Table 2.** Parameter Values by Fitting the Impedance Data of (PPV|PSS)_3_ Films to Equivalent Circuit Shown in Figure 5.

**Sample**	**Polymerization**	***R*_s_ (Ω·cm^2^)**	***R*_f_ (Ω·cm^2^)**	***R_ct_* (Ω·cm^2^)**	***C*_dl_ (μF·cm^−2^)**	***C*_f_ (μF·cm ^−2^)**
**I**	110 °C @ 2 h	120	180	250	5.1	6.1
**II**	180 °C @ 4 h	120	750	820	5.1	4.9
**III**	210 °C @ 12 h	120	2300	2900	2.5	3.2
**IV**	No heating	130	115	170	9	6.2
**V**	210 °C @ 12 h	128	20,000	24,000	5.2	5.9
